# Differences in Stride Characteristics Between Lead and Wheel Horses in Competitive Chuckwagon Racing

**DOI:** 10.3390/ani16121890

**Published:** 2026-06-18

**Authors:** Thilo Pfau, Matthijs van den Broek, Brittany L. Davis, Charlotte De Bruyne, Camille M. Eamon, Maggie Fallscheer, Sara Frostad, Karelhia Garcia-Alamo, Sara Skotarek Loch, Yuji Takahashi, Renate Weller, Zoe Y. S. Chan

**Affiliations:** 1 Faculty of Kinesiology, University of Calgary, Calgary, AB T2N 1N4, Canada; 2 Faculty of Veterinary Medicine, University of Calgary, Calgary, AB T2N 4Z6, Canada; 3 Faculty of Mechanical Engineering, Delft University of Technology, 2600 AA Delft, The Netherlands; 4 Sports Science Division, Equine Research Institute of Japan Racing Association, Shimotsuke 329-0412, Tochigi, Japan

**Keywords:** horse, stride parameters, Chuckwagon racing, lead horses, wheel horses

## Abstract

Background: In Chuckwagon racing, teams of four Thoroughbreds with two lead horses in front and two wheel horses directly behind pull a 600 kg wagon around a racetrack. Our study focuses on stride parameters that have previously retrospectively predicted impending injuries. We hypothesize that in aid of the initiation of the tight figure-of-eight turn at the start of the race, the more agile lead horses gallop with higher stride frequency (SF) and reduced stride length (SL), and that differences are exacerbated in the initial accelerative race segments. Methods: Speed, SF, and SL were quantified with global navigation satellite system (GNSS) loggers over consecutive 100 m segments over 5 furlongs in sixty horses competing in Chuckwagon racing. Mixed models for SF and SL (*p* < 0.05, Bonferroni correction) investigated the effect of speed (fixed covariate), race segment (100 m to 1000 m), and position (lead or wheel horse) as fixed factors and their two-way interactions. Results: SF and SL were significantly affected by all two-way interactions (all *p* ≤ 0.025). At the average speed of 55.5 km/h, SF was 2.333 and 2.293 Hz, SL was 6.624 and 6.735 m for lead and wheel horses, respectively. Larger SF and SL differences between lead and wheel horses were found at the start and the end of the race. Conclusions: Particularly at the start and end of a Chuckwagon race, there are distinct differences in stride parameters between lead and wheel horses. Specific speed–SF–SL models for Chuckwagon lead and wheel horses might benefit from incorporating race phase.

## Simple Summary

Chuckwagon racing involves teams of four Thoroughbreds in a 2 × 2 formation pulling a 600 kg wagon around a racetrack. We focus on differences in stride length (SL) and the number of strides taken per second (SF) between the front (lead) horses and the back (wheel) horses in relation to their distinct roles as agile (lead horses) or load pullers (wheel horses). Speed, SF, and SL were quantified with global navigation satellite system (GNSS) technology per 100 m race segment. Sixty horses competing in Chuckwagon racing at the Calgary Stampede Rangeland Derby were measured. At the average racing speed (55.5 km/h), lead horses took a higher number of strides per second compared to wheel horses. Consequently, lead horses took shorter strides to gallop at that same speed. Larger differences in stride characteristics between lead and wheel horses were found at the start and the end of the race while more similar stride characteristics were found in the middle sections of the race. The roles of body shape—lead horses are chosen for their agility, wheel horses for their pulling power—and increase/decrease in speed at beginning and end of races should be further investigated.

## 1. Introduction

In Thoroughbred racing globally, musculoskeletal injuries incurred during training and racing are a major welfare issue and a common reason for horses being withdrawn from training and racing (e.g., [1,2,3,4,5,6,7,8,9,10,11]). A recent review focusing on catastrophic fractures highlights the complex, multi-factorial nature and the complexity of practical efforts aiming at their prevention [12]. One attempt of contributing quantitative evidence to help identify horses of heightened risk of injury is the use of ‘biometric’ sensors that are designed to capture changes in movement with the aim of predicting impending musculoskeletal injuries [13,14,15]. The types of sensors used for these approaches typically measure some fundamental stride characteristics (speed, stride frequency (SF), and stride length (SL)) [14,16,17]. Optionally, these ‘biometric’ sensors can also measure higher signal frequency characteristics such as tri-axial acceleration or rate of turn, and typical traces for uninjured, well-performing horses have been utilized for comparison purposes to aid the detection of horses with impending injuries [15].

Particularly relevant for the current study is the ability of ‘biometric’ sensors to capture fundamental stride characteristics, such as speed and SL. These parameters have been shown to undergo characteristic changes over consecutive races prior to injury [13]. However, in addition to associations with impending injuries, a multitude of factors including surface type and properties, race progression, fatigue, track incline, and curvature modulate the combination of speed, SF, and SL values that can be ‘chosen’ by a horse during high-speed exercise [16,17,18,19,20,21,22]. Consequently, such changes might mask or confound injury-related changes and thus affect accuracy and precision of automated injury prediction. In the current study, the focus is on a discipline-specific quantification of speed, SF, and SL for Thoroughbred racehorses participating in Chuckwagon racing.

Chuckwagon racing is a Thoroughbred racing discipline where in contrast to Thoroughbred flat racing, unique biomechanical characteristics are encountered which we hypothesize to affect fundamental stride characteristics. First, fundamentals of physics would indicate that the horizontal forces associated with load pulling (here a 600 kg wagon) are increased when forwards or sideways velocities are undergoing larger changes. Kinematic changes related to load pulling have previously been documented in Standardbreds [23]. Second, races are held on dirt racetracks specifically prepared with a comparatively shallow cushion preventing the wagon wheels from sinking into the surface. We have shown that stride characteristics are changing with these specific surface preparations [19]. Third, the wagon-pulling horses are not carrying a rider, allowing fundamental comparisons between unridden Chuckwagon horses and horses ridden by jockeys in many other racing jurisdictions. Fourth, the comparatively short Chuckwagon racing season is very intense, with more than 50 race days over approximately three months across weekly or biweekly multiday events and races scheduled on consecutive days during these events.

The current study is specifically aimed at investigating differences between horses in the front row of the 2 × 2 configuration, known as lead horses (or leaders), and horses in the back row immediately in front of the wagon, known as wheel horses (or wheelers). The agile lead horses aid in the initiation of the tight figure-of-eight turn in the infield at the start of the race, while the wheel horses are selected for their power needed to pull the heavy wagon. We have previously shown with a validated approach for measuring speed and stride characteristics [24], that in Chuckwagon training exercises, there are complex interactions between speed and stride parameters dependent on whether horses gallop on straight or curved track sections [20]. In the current study, we hypothesize that, in Chuckwagon racing, in accordance with their position within the 2 × 2 formation, the agile lead horses will gallop with higher SF and reduced SL compared to the powerful wheel horses. In alignment with the complex interactions with track geometry, we hypothesize that differences between lead and wheel horses would be modulated by race progression and would be more exacerbated during the initial accelerative part of the race compared to the more steady-state gallop in the later parts of the race.

## 2. Materials and Methods

### 2.1. Ethical Approval

This study was approved by the University of Calgary Veterinary Sciences Animal Care and Use Committee (VSACC) with approval number AC23-0010. Drivers of Chuckwagon outfits competing at the 2025 Calgary Stampede Rangeland Derby provided written informed consent for study participation.

### 2.2. Data Collection

Drivers were recruited for study participation on a voluntary, convenience sample basis. All 27 drivers were approached for voluntary study participation of one of their Chuckwagon pulling horses in each of the ten races between 4 July and 13 July 2025. All horses participating in the study were geldings. Age and body mass of the horses were not recorded.

Horses selected for study participation were instrumented, in accordance with our previously established method described in [20], with Global Navigation Satellite System (GNSS) loggers (RaceBox Mini S, RaceBox Motorsport LLC, Altamonte Springs, FL, USA). Briefly, we attached the small (82 mm × 72 mm × 25 mm) and lightweight (106 g) device to the back pad or back strap of the horse’s harness. A maximum of ten data collection devices were available on each race day.

Data collection was initiated prior to the horses entering the track via a Bluetooth connection from dedicated iOS (iPhones, Apple Inc., Cupertino, CA, USA) or Android phones (Samsung Galaxy S22, Seoul, Korea) running the RaceBox app (RaceBox Motorsport LLC). Data collection was stopped when the outfit returned to their respective stable after the race. At that point, the GNSS records were uploaded (via the app) to the cloud and then downloaded onto a Windows (Microsoft, Redmond, WA, USA) laptop computer for analysis with custom software written in MATLAB (R2024b) (Mathworks, Natick, MA, USA). The loggers were configured to sample GNSS records at a rate of 25 Hz.

### 2.3. Data Processing

**Figure 1:**
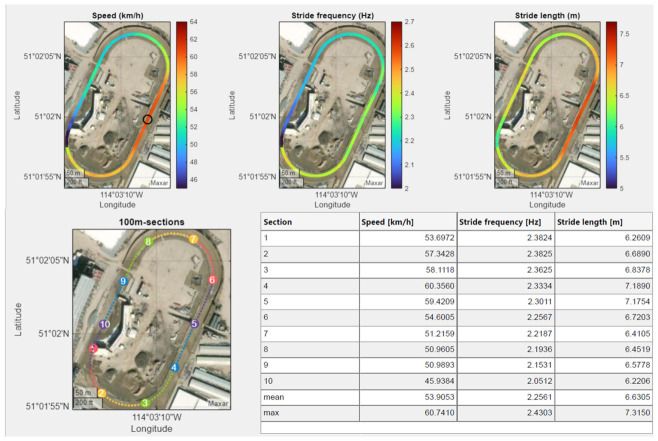
Example of GNSS-derived speed and stride parameters for ten 100 m segments along the Calgary Stampede racetrack from one example horse. Speed (top left in km/h), SF (top middle in Hz), SL (top right in m) as well as an indication of the location of the ten 100 m segments along the race progression (bottom left) are shown. Color coding (blue: low values to red: high values) is used to illustrate speed, SF, and SL. Different colors also indicate the individual segments (1 to 10) for which average values were calculated and reported in the associated data table (bottom right). The speed trace (top left) additionally indicates the location on the track where maximum speed was achieved (black circle), here approximately halfway along the back straight.

Data exported from each GNSS logger included UTC time (date and time in hours, minutes and seconds) with two decimal places, latitude and longitude (in decimal degrees) with seven decimal places, and velocity (in m/s) with two decimal places. For the current study, data processing was modified from ref. [20]. Average values of speed, SF, and SL were calculated for ten consecutive, non-overlapping 100 m segments of the 5-furlong racetrack. The first segment was initiated when the horse to which the GNSS-logger was attached had crossed a speed-threshold of 36 km/h (10 m/s) [20]. This led to consistent segments across the racetrack after the outfits had left the infield area. The example provided in Figure 1 shows the distribution of the segments, numbered from 1 to 10 for each 100 m segment across the race with segments 1 to 3 (100 m to 300 m) representing the acceleration into and through the first curve, segments 4 and 5 (400 m to 500 m) coinciding with the progression along the back straight, segments 6 to 8 (600 m to 800 m) originating from the sections through and out of the second curve and segments 9 and 10 (900 m to 1000 m) representing the final straight and across and beyond the finish line.

### 2.4. Statistical Analysis

Data were tabulated in Excel (Microsoft, Redmond, WA, USA) including information about the date of the race (4 July to 13 July 2025), driver (outfit) name, horse number (consecutive number from 1 to 60 assigned in order of occurrence of the first data point from each new horse), and position (left or right lead horse, left or right wheel horse). One record was created for each race segment (1 through 10) with values of speed (in km/h), stride frequency (in Hz), and stride length (in m). Dates were subsequently replaced by consecutive numbers for each race day (1 to 10), and driver names replaced by consecutive numbers (1 through 11).

Statistical analysis was performed in IBM SPSS Statistics (v31, IBM, Armonk, NY, USA) after importing the Excel data table into SPSS. Level of significance was chosen as *p* < 0.05 with pairwise comparisons conducted with Bonferroni correction where applicable.

In order to describe the progression of speed and stride parameters along a typical Chuckwagon race at the investigated racetrack, box plots of speed, SF, and SL were generated as a function of race segment independent of horse position.

Differences in speed, stride frequency, and stride time between lead and wheel horses were further investigated with linear mixed model analysis. Due to the study design, with instrumentation of horses during competitive racing restricted to typically one horse per outfit, data from lead and wheel horses were not collected in matched pairs, i.e., lead and wheel horses were collected from different outfits. Consequently, we first tested for differences in speed between the lead and wheel horses with a linear mixed model, applying horse as the random factor and position (lead vs. wheel) and race segment (1 through 10) as fixed factors, and the two-way interaction between position and race segment was implemented.

Subsequently, separate linear mixed models for stride frequency and stride length were implemented. These models featured horse as the random factor, with position (lead vs. wheel) and race segment (1 through 10) as fixed factors and speed as a fixed covariate. Bonferroni-corrected significance was calculated for pairwise differences between race segments. Two-way interactions between fixed factors and between each fixed factor and the fixed covariate were also entered into the model. Estimated marginal means and their 95% confidence intervals were calculated for the two-way interaction between wheel and lead horses and race segment. Model fit was evaluated by plotting histograms of model residuals overlayed onto a normal distribution fitted to the residual data. Marginal and conditional R^2^ values were calculated to illustrate the amount of variation captured by the complete model (conditional R^2^) as well as by models based on the fixed factors only (marginal R^2^).

Plots were created with race segment (1 to 10 labeled as 100 m to 1000 m) on the x-axis and raw data (either SF or SL) on the left y-axis, color-coded for data from lead horses (blue ‘o’) and wheel horses (red ‘x’). Into these plots, mixed model EMMs (with 95% confidence intervals) taken from the speed-corrected (speed as fixed covariate) models, once again color-coded for lead horses (blue dotted line) and wheel horses (red dash-dotted line), were plotted. To illustrate how the fixed speed covariate influences the analysis, we plotted speed EMM values (and 95% confidence intervals) for each segment for lead horses (blue solid line) and wheel horses (red dashed line) along the right y-axis.

As a further illustration of the mixed model analysis for SF and SL (with speed as the fixed covariate), separate plots of SF and SL were created for lead horses and wheel horses with speed on the x-axis, and SF or SL on the y-axis (Figure S1). These plots contained individual data points of all lead horses (‘o’) and wheel horses (‘x’), respectively, and color-coded by race segment—from cyan for segment 1 (100 m) to magenta for segment 10 (1000 m) (MATLAB color map ‘cool’). Into these raw data plots, lines of best fit from the mixed model coefficients were plotted for each race segment, color-coded from cyan to magenta.

## 3. Results

Across the ten days of racing, 835 data sets consisting of speed, SF, and SL for individual 100 m race segments were recorded across 60 horses from 11 Chuckwagon outfits. Table 1 lists the distribution of recordings from each outfit with information about the number of individual horses sampled per outfit over ten days of racing and their positions within the outfit.

### 3.1. Speed, Stride Frequency, and Stride Length Across 100 m Race Segments for All Horses

**Figure 2:**
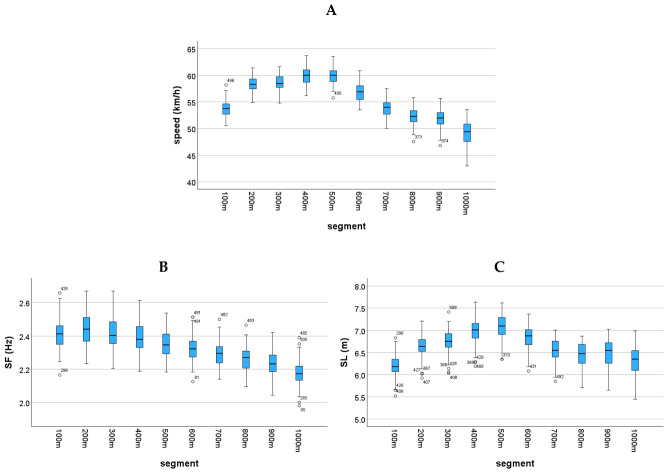
(**A**): Speed (km/h), (**B**): stride frequency (SF) (Hz), and (**C**): stride length (SL) (m) over consecutive 100 m segments from 60 Chuckwagon horses measured over ten days of racing at the 2025 Calgary Stampede Rangeland Derby.

Progression of speed, SF, and SL across the ten subsequent 100 m race segments for the 60 Chuckwagon horses collected over ten race days are illustrated in Figure 2. Both speed and SL follow similar patterns with an initial increase from the start throughout the first curve and reaching the highest values of approximately 60 km/h and 7.11 m stride length in the fifth segment around 500 m into the race along the second half of the backstretch. From then onward, both speed and SL drop markedly throughout segments 6 and 7 (600 to 700 m into the race, located along the second curve), before plateauing between 800 and 900 m into the race and further dropping for the last race segment. SF follows a different pattern. It increases to a peak value of 2.43 Hz in segment 2 (200 m into the race) and then decreases steadily throughout the remainder of the race.

Mixed model analysis with horse as random factor and race segment as fixed factor indicates significant effects of race segment for speed, SF, and SL (all *p* < 0.001) with pairwise comparisons (after Bonferroni correction) indicating significant differences between all pairings except the following segments: 1 vs. 7, 2 vs. 3, 4 vs. 5 and 8 vs. 9 for speed, 1 vs. 3 and 1 vs. 4 for SF, and 7 vs. 9 and 8 vs. 9 for SL.

### 3.2. Speed, Stride Frequency, and Stride Length Differences Between Lead and Wheel Horses

Mixed model analysis for speed indicated a significant effect of race segment (*p* < 0.001). However, no significant difference was found between lead and wheel horses (*p* = 0.107) nor for the two-way interaction (*p* = 0.883).

Mixed model analysis indicated that SF was significantly affected by all three two-way interactions (race segment × speed: *p* = 0.025; lead vs. wheel × speed: *p* < 0.001; lead vs. wheel × race segment: *p* < 0.001). SL was also significantly affected by all three two-way interactions (race segment × speed: *p* = 0.001, remaining *p* < 0.001). Speed-adjusted SF, calculated at an average speed of 55.5 km/h, was 2.333 Hz higher for lead horses than for wheel horses, which showed a speed-corrected average SF value of 2.293 Hz. Conversely, after speed correction, SL was lower for lead horses at 6.624 m than for wheel horses with an SL of 6.735 m at an average speed of 55.5 km/h.

Table 2 (SF in Hz) and Table 3 (SL in m) provide an overview of the values of the estimated fixed effects parameters and their confidence intervals. These results show evidence of increases in both SF (0.008 Hz per km/h) and SL (0.103 m per km/h) with speed, with an additional increase in SF (0.012 Hz per km/h) in combination with speed for lead horses compared to wheel horses. With increasing speed, SL increases less for lead horses (−0.032 m per km/h) compared to wheel horses. Table 2 and Table 3 also illustrate steeper increases in SF with speed and shallower increases in SL with speed for the initial race segments (see rows [100 m to 1000 m] × speed in Table 2 and Table 3).

**Figure 3:**
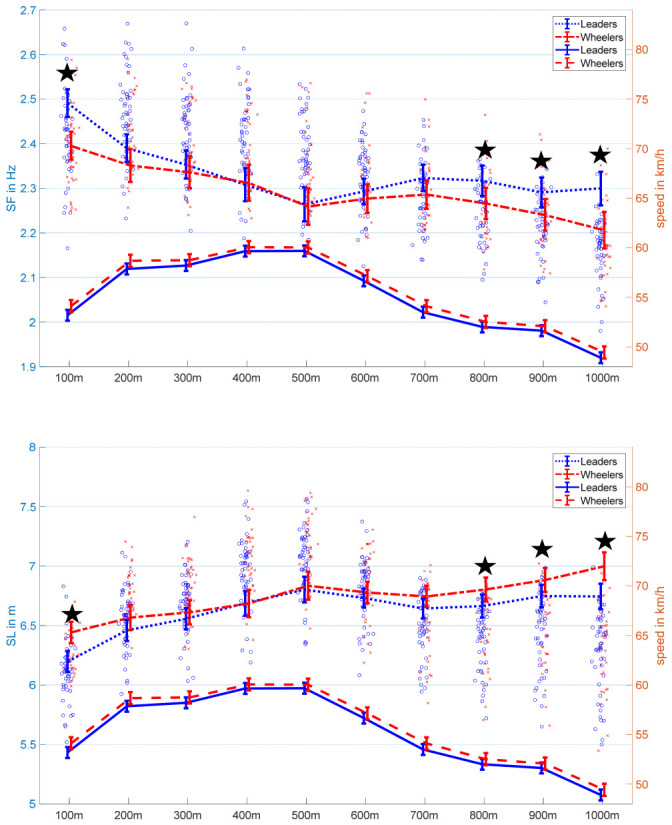
Estimated marginal means (EMMs) and 95% confidence intervals for lead horses (blue) and wheel horses (red) from mixed model analysis for speed (solid and dashed lines), SF (top panel, dotted and dash-dotted lines), and SL (bottom panel, dotted and dash-dotted lines). Speed EMM values illustrate a consistent, non-significantly different pattern between lead horses and wheel horses across race progression. Both SF and SL reveal patterns with larger differences between lead horses and wheel horses early and late in the race with closely matched values in the middle race segments, with significant differences in the first segment, and the last three segments highlighted with a ‘star’ symbol (see also Table 4 and Table 5).

Plots with EMM values (and 95% confidence intervals), calculated at the mean speed of 55.5 km/h, for SF and SL across race segments for lead horses (blue dotted) and wheel horses (red dash-dotted) are shown in Figure 3. In the same figure panels, estimated mean values (and 95% confidence intervals) for speed across all race segments separately for lead horses (blue solid line) and wheel horses (red dashed line) are plotted. In addition to the speed-corrected EMM values, raw SF and SL data are shown for lead horses (blue circles) and wheel horses (red crosses). Speed plots illustrate that wheel horses consistently have (non-significantly) higher values across all race segments compared to lead horses. Speed-corrected SF and SL values indicate higher SF and lower SL values early (through 300 m, significant at 100 m) and late (significant from 800 m to 1000 m) in the race for lead horses compared to wheel horses. EMM values (with 95% confidence intervals) for lead horses and for wheel horses are presented in Table 4 for SF and in Table 5 for SL. Segment 1 (100 m) and segment 8 (800 m) to segment 10 (1000 m) indicate significant pairwise differences (Bonferroni-corrected) between lead horses and wheel horses for both SF and SL (Table 4 and Table 5).

Supplementary Figure S1 provides further visual insights into the model estimates provided for SF and SL separately for lead horses (left panels) and wheel horses (right panels). Raw data points and linear model fits are color-coded by race segment (from cyan to magenta) with speed on the x-axis.

## 4. Discussion

This is the first study reporting GNSS-derived speed, stride frequency (SF), and stride length (SL) measured during competitive Chuckwagon racing. We employed validated algorithms [24] for calculating stride frequency from GNSS speed fluctuations recorded at a 25 Hz sample rate from commercially available GNSS loggers with built-in inertial measurement units. In this study, GNSS speed and location data were utilized. Loggers were securely mounted to the back pad of Chuckwagon racing harness, utilizing an attachment and data collection protocol that had been optimized during Chuckwagon training sessions [20] featuring minimal interference with routine racing practices.

In accordance with our first hypothesis, lead horses were found to gallop with higher SF, i.e., taking a higher number of strides per unit time and accordingly reduced SL compared to wheel horses. At the average speed of 55.5 km/h and over the typical race distance of approximately 1000 m (5 furlongs) for Chuckwagon racing, the difference in SL of 6.624 m for lead horses and 6.735 m for wheel horses resulted in approximately 2.5 strides more for the lead horses in each race, a rather small difference. It seems worth mentioning that at the beginning of the race, the lead horses are estimated to gallop with a considerably higher SF than the wheel horses at the higher end of the speed scale (see Figure S1, top panels, cyan dots and lines). This effect would slightly increase the small difference in the number of stride cycles compared to the wheel horses accumulated in each race; however, the total difference in stride cycles would remain small overall.

A fundamental limitation of our study is related to the measurements being undertaken during competitive race events. Lead and wheel horses were sampled from different Chuckwagon outfits. While this was a suboptimal study design for comparing lead to wheel horses, this was a practical necessity maximizing our ability to initiate data collection without causing time delays in the televised races at the Rangeland Derby. This required us to distribute data loggers across multiple outfits and races. As a result, lead horses and wheel horses were not as tightly matched in speed as could have been the case had they been sampled in matched pairs from the same outfit.

Consequently, we first evaluated any differences in speed between the lead and wheel horses and found no evidence of a statistically significant difference. Visually, the curve of speed values across race segments (Figure 2) for the lead horses is consistently below the curve for the wheel horses. This is interesting since our study reports significantly higher SF values for the lead horses (Figure 2, Table 4) and typically, lower speed would be associated with lower gallop SF [17,21,25]. As a comparison, we have estimated the expected amount of increase in SF and SL from published Thoroughbred flat racehorse data [21]. Based on a linear fit for SF over speed [21] and assuming an increase from a 55.24 km/h for lead horses to 55.78 km/h for wheel horses, wheel horses would be expected to gallop with a 0.006 Hz higher SF. Our study however indicates that lead horses—not wheel horses—galloped with approximately 0.04 Hz higher SF. Wheel horses would be expected to gallop with approximately 0.05 m (5 cm) longer strides based on ref. [21]. SL value differences in the present study (Table 5), with the exception of between 400 and 600 m, exceed this value of 5 cm. Further studies should aim at collecting matched data with multiple horses instrumented from within the same outfit. It would also seem important to determine whether these small differences might be associated with conformational changes—lead horses are typically selected for their “agility”—or related to the mechanics of load pulling for horses in the front and back rows.

In accordance with our second hypothesis, speed-corrected SF and SL values showed higher differences between lead and wheel horses in the initial 100 m race segment. The lead horses were galloping with higher SF and lower SL during this accelerative race phase compared to the wheel horses (Figure 2, Table 4 and Table 5). The increase in speed in this phase (between 100 m and 200 m) was, with approximately 9% (from 53.6 km/h to 58.2 km/h), comparatively modest. We had deliberately chosen a speed threshold of 36 km/h (10 m/s) that had to be exceeded to initiate data analysis to avoid including the complex figure-of-8 maneuver at the start of the race into our data analysis and we had previously piloted this approach in Chuckwagon horses in training [20]. Our study focused on the biomechanically ‘less complex’ gallop movement when the horses gallop on the track in straight lines or navigating large radius curves.

Between the first two 100 m segments, SF marginally increases from 2.420 Hz to 2.456 Hz for lead horses and from 2.369 Hz to 2.399 Hz for wheel horses, or between 1.3 and 1.5%. SL on the other hand increases more pronouncedly from 6.11 m to 6.55 m for lead horses and from 6.35 m to 6.8 m for wheel horses, or by approximately 7%. Conclusions from the data set from Takahashi and co-workers [16] for Thoroughbred flat racing sprint races on dirt tracks show a lower SF of 2.23 Hz and higher SL of 6.89 m at a speed of 15.4 m/s (55.4 km/h) during the initial race phase. From these comparative results, it appears that SF might be higher for chuckwagon horses compared with flat racehorses in Japan. However, multiple factors complicate this comparison, including surface preparation or racecourse geometry between the two disciplines. Consequently, the results of our observational study might (or might not) be useful in the context of further investigations, for example, for the development of specific prediction models for individual racing jurisdictions or disciplines.

Interestingly, our study also showed significant differences between lead and wheel horses in speed-corrected SF and SL EMM values at the end of the race (from 800 m to 1000 m). Based on our speed threshold of 36 km/h, these segments are located along the final straight, where the horses slow down, in particular after crossing the finish line. Consequently, we speculate that one of the underlying reasons for the pronounced changes in stride parameters between lead and wheel horses has to do with the mechanics of the horizontal load of a 600 kg Chuckwagon and the need to accelerate or slow down the added mass. This aspect deserves a more controlled study investigating conformational differences together with non-steady-state locomotion as potential explanatory variables for SF and SL differences.

The speed-corrected EMM differences for SL between lead and wheel horses in the initial phases (segment 1 and 2) and toward the end of the race (700 m–1000 m) show values exceeding 0.10 m (see Table 5). A reduction by a comparable value has been associated with a significantly increased risk of musculoskeletal injury over subsequent races in Thoroughbred flat racing [13]. The lead-vs.-wheel horse differences for the first and last segment (differences of 0.246 and 0.254 m, Table 5 delta values) also exceed track maintenance-related changes in SL measured on the same track on a previous occasion [19]. Furthermore, a recent study of Chuckwagon horses in training showed SL reductions of 0.136 m in curved compared to straight sections [20]. In our “in-race” study, differences in the raw data between lead and wheel horses along the final curve (Figure 2, blue circles vs. red crosses) appear to be comparatively unremarkable. These complex changes in SL and the race-segment-specific differences between wheel and lead horses in our observational study further highlight the potential need for further investigations into the complex multi-factorial relationship between speed, SF- and SL for the specific conditions encountered during training and racing for each racing discipline.

Of course, other racing disciplines do not feature distinct “positions”, such as lead and wheel horses selected for specific tasks. However, complex stride parameter changes as a function of speed, surface, and race phase have also been documented with GPS or GNSS-based approaches in flat racing [16,17]. Interestingly, flat racing stride parameters appear to change as a function of surface differently for the individual race phases, with shorter strides on dirt compared to turf only for the later race phases. It should be further investigated to what extent race position, e.g., the front runner with no space constraints versus the remaining horses that may have to adapt their strides to horses in front of them, show similar effects to the differences between lead horses and wheel horses in Chuckwagon racing. It has been speculated that different ‘roles’ (chasers versus front runners) might relate to different pacing strategies [26]. With space for three or more Chuckwagon outfits next to each other on the racetrack, the effect of chasers versus front runners might have less of an influence between outfits; however, race strategy should also be taken into account in future studies.

## 5. Conclusions

Our study highlights the complex nature of fundamental movement adaptations such as the association between speed, stride frequency, and stride length for a specific racing discipline. Differences between horses performing distinct tasks exhibit differences in stride characteristics. It is very interesting that differences between horses used in specific positions in front of the heavy wagon are more or less pronounced over the course of the race and in association with mixed model analysis, with speed as the covariate.

Speed is very generally associated with the magnitude of (vertical) force with increasing speed, resulting in a lower duty factor and consequently a higher peak force across straight lines, curved, and inclined gallops [21,22,27,28]. With speed approximating the force magnitude and with SF (or SL) approximating the number of stimuli over time (over distance), the triad of speed, SF, and SL might prove useful for further improving our understanding of the development of overuse injuries. Our study should be seen as a first attempt at quantifying stride characteristics in association with discipline-specific factors, such as the position of the horse within a team of horses and race progression for the discipline of Chuckwagon racing. Further follow-up studies are needed, combining in-race measurements with a number of confounding variables, such as quantitative surface characteristics and individualized information, for example, conformational characteristics in a matched design with simultaneous sampling from multiple horses within each team.

## Figures and Tables

**Table 1:** Distribution of GNSS recordings collected from Chuckwagon horses during ten days of competitive racing. Data were obtained from 60 individual horses belonging to 11 Chuckwagon outfits. A total of 84 (including 22 LL: left lead, 26 RL: right lead, 18 LW: left wheel and 18 RW: right wheel) recordings were analyzed. Each recording consisted of multiple 100 m sections along the racetrack from which speed, stride frequency, and stride length were derived. The table summarizes the number of horses and recordings contributed by each outfit, together with the distribution of recording positions.

Outfit #	Horses	Recordings	Recordings by Position
1	5	8	LL: 3; RL: 0; LW: 0; RW: 5
2	8	8	LL: 2; RL: 0; LW: 4; RW: 2
3	5	10	LL: 0; RL: 5; LW: 5; RW: 0
4	5	9	LL: 7; RL: 2; LW: 0; RW: 0
5	1	1	LL: 0; RL: 0; LW: 0; RW: 1
6	6	9	LL: 4; RL: 3; LW: 0; RW: 2
7	2	2	LL: 0; RL: 2; LW: 0; RW: 0
8	7	11	LL: 4; RL: 1; LW: 4; RW: 2
9	8	8	LL: 0; RL: 2; LW: 4; RW: 2
10	6	9	LL: 0; RL: 7; LW: 0; RW: 2
11	7	9	LL: 2; RL: 4; LW: 1; RW: 2
**Total**	**60**	**84**	**LL: 22; RL: 26; LW: 18; RW: 18**

**Table 2:** Estimates of fixed effect parameters for linear mixed model analysis for SF (in Hz). Fixed effects: “Lead/Wheel”: for lead and wheel horses; “100 m to 1000 m”: for 100 m race segments; “speed”: fixed covariate speed in km/h. One-way and two-way effects and values for lower and upper bound of 95% confidence intervals are provided.

Parameter	Estimate	Lower Bound	Upper Bound
Intercept	1.740	1.512	1.969
Lead	−0.580	−0.803	−0.357
Wheel	ref	.	.
100 m	−0.430	−0.806	−0.053
200 m	−0.227	−0.600	0.146
300 m	−0.101	−0.440	0.237
400 m	0.082	−0.274	0.438
500 m	−0.111	−0.478	0.257
600 m	−0.075	−0.399	0.250
700 m	0.117	−0.210	0.445
800 m	0.150	−0.174	0.474
900 m	0.182	−0.103	0.467
1000 m	ref	.	.
speed	0.008	0.004	0.013
Lead × 100 m	0.002	−0.029	0.033
Lead × 200 m	−0.054	−0.101	−0.008
Lead × 300 m	−0.076	−0.123	−0.028
Lead × 400 m	−0.098	−0.150	−0.045
Lead × 500 m	−0.088	−0.141	−0.036
Lead × 600 m	−0.077	−0.119	−0.036
Lead × 700 m	−0.056	−0.087	−0.025
Lead × 800 m	−0.042	−0.069	−0.016
Lead × 900 m	−0.043	−0.069	−0.017
Lead × 1000 m	ref	.	.
Wheel × [segment]	ref	.	.
Lead × speed	0.012	0.008	0.017
Wheel × speed	ref	.	.
100 m × speed	0.011	0.004	0.018
200 m × speed	0.007	0.000	0.013
300 m × speed	0.004	−0.002	0.010
400 m × speed	0.000	−0.006	0.007
500 m × speed	0.003	−0.003	0.009
600 m × speed	0.003	−0.003	0.009
700 m × speed	−0.001	−0.007	0.006
800 m × speed	−0.002	−0.008	0.005
900 m × speed	−0.003	−0.008	0.003
1000 m × speed	ref	.	.

**Table 3:** Estimates of fixed effect parameters for linear mixed model analysis for SL (in meters). Fixed effects: “Lead/Wheel”: for lead and wheel horses; “100 m to 1000 m”: for 100 m race segments; “speed”: fixed covariate speed in km/h. One-way and two-way estimates and values for lower and upper bound of 95% confidence interval are provided.

Parameter	Estimate	Lower Bound	Upper Bound
Intercept	1.278	0.619	1.937
Lead	1.528	0.885	2.171
Wheel	ref	.	.
100 m	1.446	0.359	2.532
200 m	1.269	0.192	2.345
300 m	0.933	−0.045	1.910
400 m	0.455	−0.573	1.482
500 m	0.854	−0.207	1.914
600 m	0.794	−0.141	1.729
700 m	0.017	−0.927	0.962
800 m	−0.127	−1.061	0.807
900 m	−0.316	−1.138	0.507
1000 m	ref	.	.
speed	0.103	0.090	0.116
Lead × 100 m	0.008	−0.080	0.097
Lead × 200 m	0.147	0.013	0.282
Lead × 300 m	0.200	0.063	0.336
Lead × 400 m	0.256	0.104	0.408
Lead × 500 m	0.222	0.071	0.374
Lead × 600 m	0.208	0.090	0.327
Lead × 700 m	0.153	0.064	0.242
Lead × 800 m	0.114	0.037	0.191
Lead × 900 m	0.119	0.044	0.193
Lead × 1000 m	ref	.	.
Wheel × [segment]	ref	.	.
Lead × speed	−0.032	−0.045	−0.019
Wheel × speed	ref	.	.
100 m × speed	−0.036	−0.057	−0.016
200 m × speed	−0.031	−0.050	−0.012
300 m × speed	−0.024	−0.041	−0.006
400 m × speed	−0.014	−0.032	0.004
500 m × speed	−0.018	−0.037	0.000
600 m × speed	−0.018	−0.035	−0.001
700 m × speed	−0.005	−0.023	0.013
800 m × speed	−0.001	−0.019	0.017
900 m × speed	0.004	−0.012	0.020
1000 m × speed	ref	.	.

**Table 4:** Estimated marginal mean (EMM) values, 95% confidence intervals, and Bonferroni-corrected pairwise *p*-values and pairwise differences between lead- and wheel-horse EMM values (delta) for SF (Hz) across race segments from mixed model analysis with speed as the fixed covariate, and race segment and lead vs. wheel horse as fixed factors, and all two-way interactions included. Marginal and conditional R^2^ values were 0.558 and 0.910.

	Lead Horses			Wheel Horses			Comparison	
Segment	EMM ^1^	Lower Bound	Upper Bound	EMM ^1^	Lower Bound	Upper Bound	*p*-Value	Delta
1	2.491 *	2.460	2.522	2.395 *	2.364	2.427	**<0.001**	0.096
2	2.390	2.359	2.421	2.351	2.314	2.388	0.077	0.039
3	2.353	2.322	2.385	2.336	2.300	2.372	0.422	0.017
4	2.308	2.271	2.345	2.312	2.272	2.353	0.850	0.004
5	2.264	2.226	2.302	2.259	2.218	2.300	0.833	0.005
6	2.293	2.264	2.321	2.277	2.245	2.309	0.452	0.016
7	2.323	2.294	2.353	2.286	2.254	2.317	0.080	0.037
8	2.317 *	2.283	2.351	2.266 *	2.231	2.301	**0.022**	0.051
9	2.291 *	2.257	2.324	2.240 *	2.205	2.276	**0.025**	0.051
10	2.300 *	2.262	2.337	2.206 *	2.165	2.247	**<0.001**	0.094

^1^ EMM values calculated at the average speed of 55.5 km/h. * Segments with Bonferroni-corrected significant differences (*p* < 0.05) between lead horses and wheel horses; associated EMM values and *p*-values in bold. Each segment represents average values calculated over 100 m of gallop from segment 1 (first 100 m) to segment 10 representing the last 100 m of the total 1000 m covered.

**Table 5:** Estimated marginal mean (EMM) values, 95% confidence intervals, and Bonferroni-corrected pairwise *p*-values and pairwise differences between lead- and wheel-horse EMM values (delta) for SL (m) across race segments from mixed model analysis, with speed as the fixed covariate, and race segment and lead vs. wheel horse as fixed factors, and all two-way interactions included. Marginal and conditional R^2^ values were 0.647 and 0.926.

	Lead Horses			Wheel Horses			Comparison	
Segment	EMM ^1^	Lower Bound	Upper Bound	EMM ^1^	Lower Bound	Upper Bound	*p*-Value	Delta
1	6.197 *	6.109	6.284	6.443 *	6.353	6.532	**<0.001**	0.246
2	6.460	6.372	6.549	6.567	6.462	6.671	0.086	0.107
3	6.557	6.468	6.646	6.611	6.510	6.713	0.381	0.054
4	6.688	6.583	6.792	6.686	6.570	6.801	0.976	−0.002
5	6.803	6.695	6.911	6.835	6.718	6.952	0.626	0.032
6	6.732	6.652	6.812	6.778	6.687	6.868	0.442	0.046
7	6.644	6.560	6.727	6.745	6.656	6.834	0.092	0.101
8	6.664 *	6.568	6.759	6.804 *	6.703	6.904	**0.026**	0.140
9	6.748 *	6.654	6.842	6.883 *	6.783	6.984	**0.033**	0.135
10	6.745 *	6.637	6.852	6.999 *	6.882	7.116	**<0.001**	0.254

^1^ EMM values calculated at the average speed of 55.5 km/h. * Segments with significant differences (*p* < 0.05) between lead horses and wheel horses; associated EMM values and *p*-values in bold.

## Data Availability

The data set associated with this manuscript has been published on figshare https://doi.org/10.6084/m9.figshare.31258633.

## Supplementary Materials

The following supporting information can be downloaded at https://www.mdpi.com/article/10.3390/ani16121890/s1, Figure S1: Scatter plots and lines of best fit from mixed model analysis for speed (x-axis) and SF (top row) or SL (bottom row) along the y-axis. Panels on the left for lead horses (“Leaders”), and wheel horses (“Wheelers”) in the panels on the right. Individual data points are color-coded by the race segment from which they originate, starting with cyan (first 100 m segment) through to magenta (last 100 m segment), utilizing the MATLAB colormap “cool”. Lines of best fit for each 100 m segment are plotted with the same color coding. Figure S2: Histogram and normal distribution fitted to the residual data for the mixed linear model residuals for SF. Horse was used as the random factor, position (lead vs. wheel) and race segment (1 through 10) as fixed factors, and speed as the fixed covariate. Pairwise two-way interactions between fixed factors and between each fixed factor and the fixed covariate (speed) were also entered into the model. Figure S3: Histogram and normal distribution fitted to the residual data for the mixed linear model residuals for SL. Horse was used as the random factor and position (lead vs. wheel), race segment (1 through 10) as fixed factors, and speed as the fixed covariate. Pairwise two-way interactions between fixed factors and between each fixed factor and the fixed covariate (speed) were also entered into the model.

## Abbreviations

The following abbreviations are used in this manuscript:

SF	Stride frequency
SL	Stride length
GNSS	Global navigation satellite system
GPS	Global positioning system
UTC	Universal time constant
