# Supplementary material for: Differences in Stride Characteristics Between Lead and Wheel Horses in Competitive Chuckwagon Racing
Source: Animals (Basel). 2026 Jun 18;16(12):1890. doi: 10.3390/ani16121890 (PMC13295957; doi:10.3390/ani16121890)
Supplement: Supplementary file 1 [file animals-16-01890-s001.zip › animals-4346657-supplementary.pdf]

Supplementary Materials

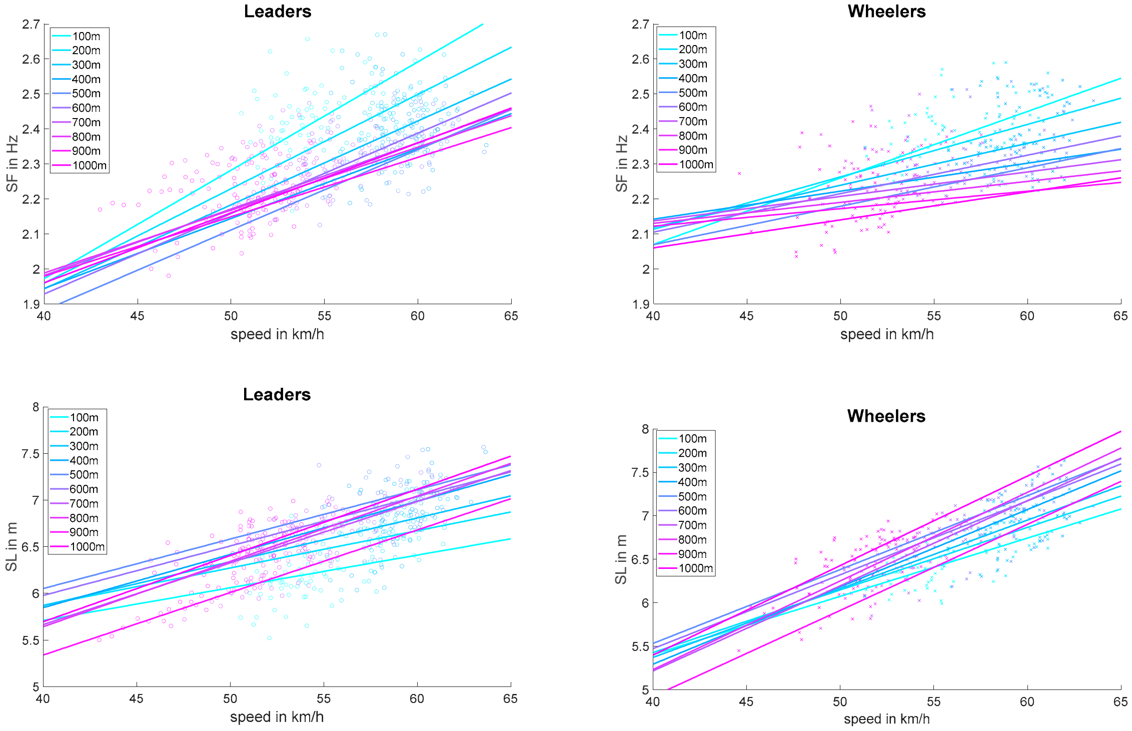

Figure S1: Scatter plots and lines of best fit from mixed model analysis for speed (x-axis) and SF (top row) or SL (bottom row) along the y-axis. Panels on the left for lead horses ("Leaders"), and wheel horses ("Wheelers") in the panels on the right. Individual data points are color-coded by the race segment from which they originate, starting with cyan (first 100 m segment) through to magenta (last 100 m segment), utilizing the MATLAB colormap "cool". Lines of best fit for each 100 m segment are plotted with the same color coding.

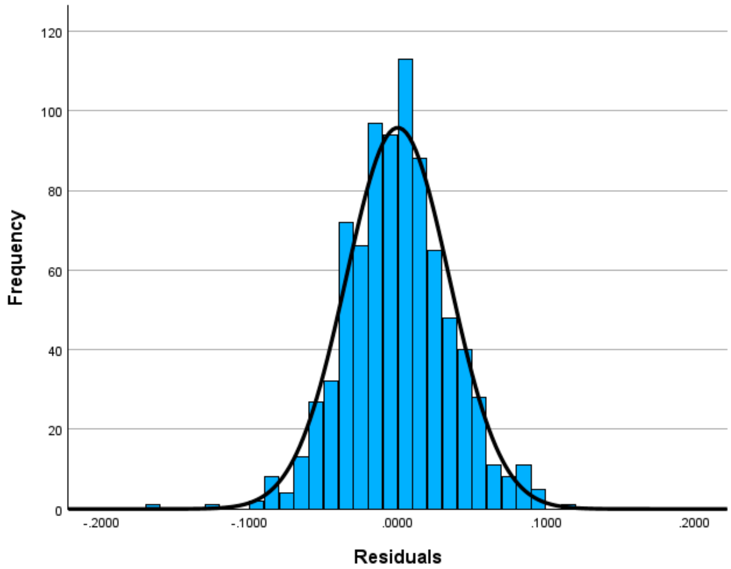

Figure S2: Histogram and normal distribution fitted to the residual data for the mixed linear model residuals for SF. Horse was used as the random factor, position (lead vs. wheel) and race segment (1 through 10) as fixed factors, and speed as the fixed covariate. Pairwise two-way interactions between fixed factors and between each fixed factor and the fixed covariate (speed) were also entered into the model.

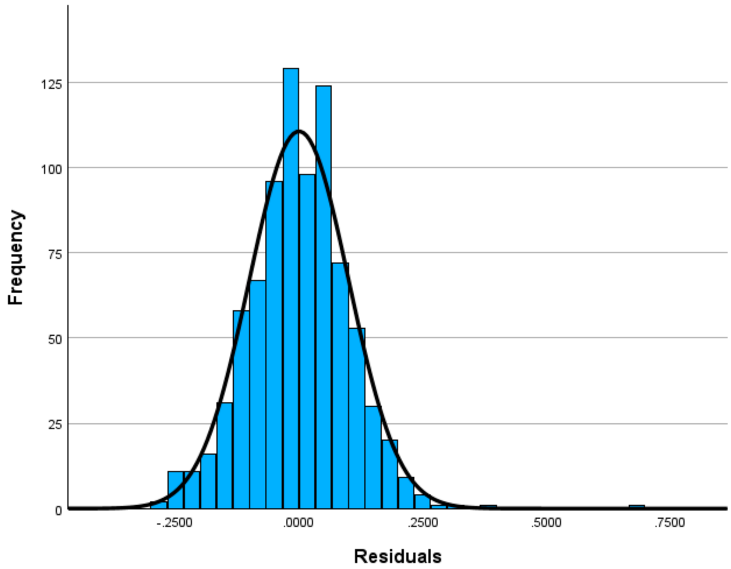

Figure S3: Histogram and normal distribution fitted to the residual data for the mixed linear model residuals for SL. Horse was used as the random factor and position (lead vs. wheel), race segment (1 through 10) as fixed factors, and speed as the fixed covariate. Pairwise two-way interactions

between fixed factors and between each fixed factor and the fixed covariate (speed) were also entered into the model.
